# Complete heart block in a Caucasian woman with Behçet’s disease: a case report

**DOI:** 10.1186/s13256-016-0890-y

**Published:** 2016-04-19

**Authors:** Sabeeh-Ur-Rehman Butt, Julian McNeil

**Affiliations:** 1 Department of Medicine, Lyell McEwin Hospital, Elizabeth Vale, SA 5112 Australia; 2 Department of Rheumatology, Modbury Hospital, Modbury, SA 5092 Australia; 3 Present Address: Ballarat Base Hospital, Drummond Street North, Ballarat Central, VIC 3350 Australia

**Keywords:** Behçet’s disease, Caucasian, Female, Heart block

## Abstract

**Background:**

Behçet’s disease is a progressive diffuse inflammatory vasculitis characterized by recurrent oral and genital ulceration and ocular inflammation. Cardiac involvement is a rare but well-documented manifestation of Behçet’s disease. Complete heart block in non-Caucasian populations has been reported previously; however, in this report, we describe a unique case of complete heart block in a Caucasian woman with Behçet’s disease.

**Case presentation:**

A 48-year-old Caucasian woman presented to our hospital with symptomatic complete heart block requiring a pacemaker implant on a background of recurrent oral and genital ulcers and oligoarthritis of 10 months’ duration. She also had a history of recurrent diarrhea with a single episode of ocular inflammation in the recent past. She had no evidence of cardiac ischemia, and her autoimmune antibodies were within normal ranges. She was diagnosed with Behçet’s disease according to international study group criteria and was commenced on prednisolone and sulfasalazine, to which she responded very well.

**Conclusions:**

Cardiac complications should be considered when making a diagnosis of Behçet’s disease, even in Caucasian patients. While mucocutaneous ulceration is indeed the most common manifestation of Behçet’s disease, cardiovascular involvement tends to cause the most morbidity and mortality.

## Background

Behçet’s disease (BD) is a progressive, diffuse vasculitis with recurrent oral and genital ulceration and ocular inflammation. Cardiac involvement, such as pericarditis, myocardial infarction, coronary arteritis, valvular regurgitation, and impaired conduction, arising with BD is a rare but well-documented manifestation [1]. However, only a handful cases of BD with complete heart block in non-Caucasian populations have been reported previously [2, 3]. This report is therefore unique in its description of a case of a Caucasian woman with BD and complete heart block.

## Case presentation

**Fig. 1:**
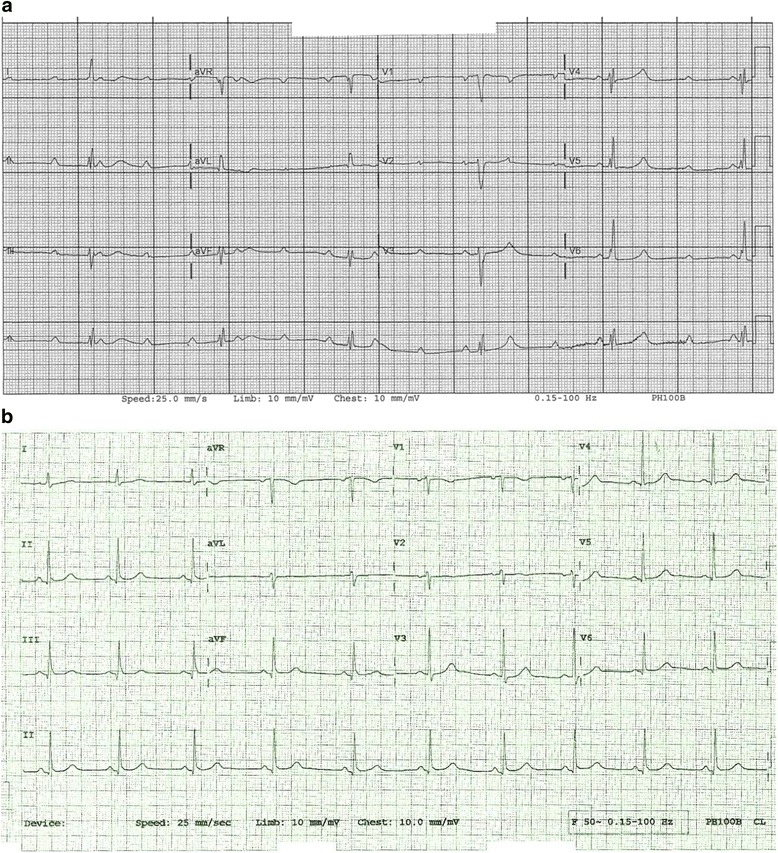
**a** Complete atrioventricular block. **b** Normal sinus rhythm

A 48-year-old Caucasian woman was admitted to our hospital with presyncope, nausea, and palpitations. An electrocardiogram (ECG) showed new, complete atrioventricular (AV) block (Fig. 1a). A permanent pacemaker was implanted, which resulted in resolution of her symptoms. Her serum troponin levels were within normal range. She subsequently developed intermittent watery diarrhea without blood or mucus and was passing 10–15 bowel movements per day. The patient reported having painful, recurrent oral and genital ulcers for the previous 10 months. She also had intermittent large joint swelling and tenderness during this period. Furthermore, she had had an episode of redness of her eyes 2 months before her first admission that had lasted for 2 weeks, consistent with uveitis. She had been admitted 3 weeks before her current presentation with chest pain due to pericarditis. An ECG at that time showed normal sinus rhythm (Fig. 1b), while an echocardiogram showed small pericardial effusion. At that time, tender swelling of her right knee joint was observed. Her physical examination also revealed numerous mucosal ulcers in her mouth and on the labia minora. Her past medical history included 40 pack-years smoking and a right lower limb deep venous thrombosis when she was pregnant with her second child.

Her clinical examination did not reveal any evidence of extraintestinal manifestation of inflammatory bowel disease. Of note, her pathergy test result was negative. The results of her autoimmune screen, including rheumatoid factor, antinuclear antibody, anti-double-stranded DNA, anti-cyclic citrullinated peptide antibody, and extractable nuclear antibodies, were negative. Her human leukocyte antigen (HLA)-B51 was negative, but she had genotype HLA-B35 in her blood. Stool microscopy and culture did not reveal any pathogens. An endoscopic evaluation showed a large ulceration in the terminal ileum that did not appear typical of Crohn’s disease. Her histopathological examination showed changes consistent with vasculitis but no granulomas or transmural inflammation. A computed tomographic enterogram showed no other areas of ulceration or evidence of Crohn’s disease. On the basis of this patient’s oral and genital ulceration, documented evidence of synovitis, vasculitic ulcer in the gastrointestinal tract, and an episode of ocular inflammation and no evidence of another inflammatory disease, a diagnosis of BD was made. She was commenced on prednisolone 50 mg with a slow tapering course, as well as sulfasalazine 500 mg twice daily that was later increased to 1 g twice daily, to which she responded with resolution of her diarrhea and mucosal ulceration. She continued to be in remission during once-monthly and then thrice-monthly clinical follow-up.

## Discussion

BD is a multisystem inflammatory vasculitis with an unclear etiopathogenesis [4]. Our patient fulfilled international criteria for BD with oral, genital, and ocular involvement, along with arthritis and cardiovascular inflammation in the presence of intestinal inflammation, while an alternative diagnosis was absent [5]. A positive pathergy test, although specific, is not sensitive. BD is a universal disorder with a greater incidence and prevalence in the regions along the Silk Road, and it occurs more often in males than in females [4, 6].

Cardiac manifestations in BD are rare but well documented and include endocarditis, pericarditis, and, less commonly, myocardial infarction, myocarditis, or vascular inflammation often involving both large and small arteries [7]. BD with complete heart block in non-Caucasian populations has been reported previously [2, 3, 7], rendering our present case report unique in its description of complete heart block in a Caucasian woman with BD.

Although the etiology of the cardiac involvement is not known, it has been suggested that inflammation affecting the conduction system leads to impairment of conduction [7, 8]. In our patient, ischemia was unlikely in the presence of normal serum troponin levels and a structurally well-preserved myocardium visualized on her echocardiogram. Preexisting congenital AV block was ruled out on the basis of the normal ECG on her first admission, when she had cardiac inflammation in the form of pericarditis. Hence, the mechanism of the conduction block in our patient was likely to be an inflammatory involvement of the conducting pathways, especially in the presence of other systemic inflammatory symptoms. HLA-B51 was absent; however, genotyping of HLA-B35 that was present in our patient has a less significant but positive association with BD [9].

## Conclusions

Cardiac inflammation due to BD may manifest as symptomatic complete heart block requiring intervention, even when the patient has a mild and readily treatment-responsive disease elsewhere. Cardiac complications should be considered when making a diagnosis of BD, even in Caucasian patients, as although mucocutaneous ulceration is indeed the most common manifestation of BD, cardiovascular involvement tends to cause the most morbidity and mortality [10].

## Consent

Written informed consent was obtained from the patient for publication of this case report and any accompanying images. A copy of the written consent is available for review by the Editor-in-Chief of this journal.

## Abbreviations

AV: atrioventricular
BD: Behçet’s disease
ECG: electrocardiogram
HLA: human leukocyte antigen
